# Global constitutionalism, applied to global health governance: uncovering legitimacy deficits and suggesting remedies

**DOI:** 10.1186/s12992-016-0216-2

**Published:** 2016-12-03

**Authors:** Gorik Ooms, Rachel Hammonds

**Affiliations:** 1 Department of Global Health and Development, London School of Hygiene and Tropical Medicine, Keppel St, London, WC1E 7HT UK; 2 Law and Development Research Group, Faculty of Law, University of Antwerp, Venusstraat 23, 2000 Antwerpen, Belgium

**Keywords:** Global health governance, Global constitutionalism, Health security, Right to health

## Abstract

**Background:**

Global constitutionalism is a way of looking at the world, at global rules and how they are made, as if there was a global constitution, empowering global institutions to act as a global government, setting rules which bind all states and people.

**Analysis:**

This essay employs global constitutionalism to examine how and why global health governance, as currently structured, has struggled to advance the right to health, a fundamental human rights obligation enshrined in the International Covenant on Economic, Social and Cultural Rights. It first examines the core structure of the global health governance architecture, and its evolution since the Second World War. Second, it identifies the main constitutionalist principles that are relevant for a global constitutionalism assessment of the core structure of the global health governance architecture. Finally, it applies these constitutionalist principles to assess the core structure of the global health governance architecture.

**Discussion:**

Leading global health institutions are structurally skewed to preserve high incomes countries’ disproportionate influence on transnational rule-making authority, and tend to prioritise infectious disease control over the comprehensive realisation of the right to health.

**Conclusion:**

A Framework Convention on Global Health could create a classic division of powers in global health governance, with WHO as the law-making power in global health governance, a global fund for health as the executive power, and the International Court of Justice as the judiciary power.

## Background

Global constitutionalism is a school of thought in international law and international relations studies. According to Falk, global constitutionalism is about the “extension of constitutional thinking to world order” [1]. The “familiar solution”, or so argues Falk, would be “the establishment of a world government with centralized institutions equipped with coercive machinery”. But it would be a mistake “to reduce the perspective of global constitutionalism to governmental alternatives to the state system”: [1] constitutional thinking can be applied to global governance that does not take the form of a global government. Global health governance, defined by Fidler as “the use of formal and informal institutions, rules, and processes by states, intergovernmental organizations, and nonstate actors to deal with challenges to health that require cross-border collective action to address effectively”, [2] includes several institutions that have been given (or have captured) the power to set norms that are either binding, or difficult to disregard, for many states. In recent years, several comprehensive studies of global health governance have been published; [3–5] they contain many critical comments, and rightfully so, in our opinion. However, in the absence of a consensual or widely shared opinion as to what global health governance *should* do, critical comments regarding how and why it is failing are both less forceful and less persuasive than they could be. This is why we think global constitutionalism, used as an analytical lens, could be helpful, in addressing the question of whether global health governance lives up to basic constitutional principles. As far as we know, global constitutionalism analysis has not yet been applied to global health governance, a study by Hawkins and Howe on the impact of bilateral trade agreements on global health excepted [6].

To avoid confusion, it is worth noting that there is a difference between global *constitutionalisation* and global *constitutionalism*. Global constitutionalisation refers to the gestation of a system of institutions that have been given (or captured) rule-making authority at a level above the state. Global constitutionalism critically examines that gestational process, looking for its successes and failures in living up to the minimum standards one would expect from a global constitutional order. In that respect, we do not entirely agree with Hawkins and Howe when they describe global constitutionalism as an “approach to governance which sees ‘constitutionalisation’ of the global sphere as a necessary and positive process” [6]. Global constitutionalism also “uncovers legitimacy deficits” in the process of global constitutionalisation, and “suggests remedies” [7]. Indeed, many global constitutionalism scholars share a feeling of discontentment about the ‘Westphalian’ world order of sovereign and independent states, resulting in a state of anarchy-absence of authority-at the global level, and are therefore inclined to view steps away from that anarchy with a benevolent eye. But they are aware that the thirst for ‘global rule of law’ risks “validating” undemocratic global institutions and “promoting dangerously seductive “over-expectations” [8]. In any case, our main interest in a global constitutionalism analysis lies in its potential to allow “extreme inequality in the world to be not only shown but also condemned” [9].

This essay first examines the core structure of the global health governance architecture, and its evolution since the Second World War. Second, it identifies the main constitutionalist principles that are relevant for a global constitutionalism assessment of the core structure of the global health governance architecture. Finally, it applies these constitutionalist principles to assess the core structure of the global health governance architecture.

## Analysis

### I. The core structure of the global health governance architecture

Applying a global constitutionalism analysis to global health governance requires that we first define what we mean with global health governance. We start from Fidler’s definition, already mentioned above, according to which global health governance “refers to the use of formal and informal institutions, rules, and processes by states, intergovernmental organizations, and nonstate actors to deal with challenges to health that require cross-border collective action to address effectively” [2]. However, for a global constitutionalism analysis, we are concerned only with institutions that have received or captured some authority to make rules that supersede national rules, and therefore our analysis excludes private for-profit and not-for-profit actors and governmental agencies that engage chiefly in bilateral agreements. In line with Youde’s and Harman’s books on global health governance, [3, 4] our analysis focuses on two leading intergovernmental organisations, the World Health Organization (WHO) and the World Bank, and the so-called new global health actors: the global health initiatives (GHIs) that have popped up since the turn of the millennium. For the sake of parsimony, our analysis will focus on the Global Fund to fight AIDS, Tuberculosis and Malaria (Global Fund).

In 1946, WHO was created and mandated to promote “the attainment by all peoples of the highest possible level of health”, and to work to “control of disease, especially communicable disease” [10]. WHO was accorded the power to elaborate international law to achieve both elements of its broad mandate. However, it has exercised its legislative powers mainly in relation to the so-called health security purpose: the International Sanitary Regulations of 1951, revised and renamed International Health Regulations (IHR) in 1969, and again revised in 2005 [11]. WHO’s most ambitious plan for promoting equitable access to essential healthcare, the practical expression of its mandate to ensure the highest possible level of health for all, was the Global Strategy for Health for All by the Year 2000, [12] based on the 1978 Declaration of Alma-Ata [13]. This strategy, however, was not translated into international law.

The Global Strategy for Health for All by the Year 2000 upset the USA and some other powerful states, which turned their back on WHO and started relying on the World Bank for global health governance [14]. The “greater funding power” of the World Bank allowed it to displace the WHO as the major influence behind health policy in poor countries [15]. Even before the Declaration was ‘translated’ into the Global Strategy for Health for All by the Year 2000-in 1981-some scholars had proposed “selective primary health care” as an “interim strategy” [16]. The World Bank never formally endorsed *selective* Primary Health Care (PHC) as its preferred strategy. However, World Bank financial support to developing countries has always come with conditions, and in the 1980s, these conditions became known as ‘structural adjustment’, which included conditions on (reducing) public social expenditure. Several scholars argue that Structural Adjustment Programmes (SAPs) thus forced developing countries to accept the selective PHC approach [17, 18]. SAPs are also blamed for pushing developing countries towards excessive reliance on ‘user fees’ or charges for health care services. Formally introduced by the 1987 Bamako Initiative, adopted by the WHO’s African regional organisation, user fees were intended to deal “with the severe economic crises facing sub-Saharan Africa, the negative effects of adjustment programmes on health, and the reluctance of donors to continue to fund recurrent costs of primary health care programmes” [19].

The publication in 1993 of the World Bank’s World Development Report ‘Investing in Health’ “reflected a marked change in the orientation of how healthcare services in resource-poor countries would be delivered” [20]. In this report, the World Bank introduced a separation between “public health”, “essential clinical services”, and “the rest of the health system” [21]. While the report clearly recommended increasing public investment in public health measures-like selective PHC, all focused on infectious disease control-and clearly recommended that “the rest of the health system” be financed privately, it was ambiguous on the financing of “essential clinical services”, referring to the early successes of the Bamako Initiative as an alternative option to public financing. The World Bank’s health sector reform strategy brought together elements of selective PHC, SAPs and the Bamako Initiative. According to Gostin, the World Bank’s ‘Investing in Health’ report “marked the zenith of its global health influence, promoting reforms meant to improve equity, efficiency and effectiveness” [22].

By the end of the 1990s, the flaws in the World Bank’s health sector strategy became apparent, particularly in low-income countries, which proved unable-or insufficiently resourced-to address the growing pandemic of HIV/AIDS effectively, or to integrate immunisation programs, previously financed by the international community, within their health systems [23]. The ‘financial slimming’ exercise of the SAPs proved to be problematic, not only from an equitable access to essential care perspective, but also from a health security perspective. The turn of the millennium saw a proliferation of GHIs, also known as public-private partnerships (PPPs) which sought largely to address communicable diseases. According to Harmer and Buse, they were “a nascent experiment in global health” in the late 1990s, but “now they are part of mainstream global health discourse and a dominant model for cooperation in a complex world” [24]. If “greater funding power” is what allowed the World Bank to displace the WHO as the major influence behind health policy in poor countries, as Abbasi argues, [15] then it should be noted that two GHIs have caught up with or even surpassed the World Bank in funding power: the Global Alliance for Vaccines and Immunisation (GAVI) and the Global Fund [25].

Global health governance continued to evolve after the creation of the Global Fund and GAVI. Every year, new GHIs are created, while few cease operations. In Hill’s words, global health governance is, and will remain, a complex adaptive system [26]. Nevertheless, we would argue that the combination of WHO, the World Bank, and the Global Fund (as an example of a major GHI) provides a representative sample of the core structure of the global health governance architecture, sufficient at least for an initial global constitutionalism assessment.

### II. Global constitutionalism (constitutionalist principles, applicable at the global level, relevant for global health governance)

Having demarcated the core structure of the global health governance architecture as the subject of our analysis, we need to clarify the constitutionalist principles we will use for our analysis. Unfortunately, there is no widely accepted definition of global constitutionalism. Besson writes (about international constitutionalism): “Promoted since the 1930s in Europe and rediscovered in the 1990s, it has meant different things to different people, has been promoted for very different reasons, and has also been criticized on many different grounds” [27]. Werner argues: “The vocabulary of constitutionalism has been used in different contexts and for different purposes, varying from in-depth critiques of existing international law to attempts to explain the rise of international tribunals, the revitalisation of international organisations, the self-understanding of European organisations in terms of constitutionalism or the development of a core of fundamental values in international law” [28]. However, in spite of this lack of consensus, there are three themes, or constitutionalist principles, that are common to most global constitutionalism analysis and research, and that seem relevant for our assessment of the core structure of the global health governance architecture.

- The degree to which rule-making authority is received or captured by global institutions is important to distinguish between ‘ordinary’ cooperation between states-presumably based on voluntary cooperation between equal, sovereign states, the kind of cooperation that Oye would call “cooperation under anarchy” [29] – and a new kind of cooperation under which states are, at times, obliged to follow the rules set at the global level, i.e., the process known as global constitutionalisation.
- The democratic legitimacy of the institutions to which rule-making power is conferred is important to assess whether the loss of sovereignty that occurs under global constitutionalisation is being ‘compensated’-or not-by genuine participation in the decision-making processes.
- Human rights law, or the degree to which human rights are realised through rules set by global institutions, is used to assess the quality of the rules set by global institutions: like national rules that violate the basic rights enshrined in the constitution are called ‘unconstitutional’, global rules that violate international human rights law can be called ‘un-global-constitutional’.

With regards to rule-making authority, most global constitutionalism analyses focus on global institutions that have received a more or less formal mandate to elaborate, promulgate or implement international law. The purpose of that focus is to distinguish between trans-national arrangements that continue to rely on voluntary cooperation between equal sovereign states-because states can at any moment step in or step out of the arrangement-and arrangements under which states give up at least part of their sovereignty-because once they adhere to the arrangement, they cannot easily withdrawn, and therefore at times decisions will be taken without their consent. Only the latter form of arrangements is of interest for global constitutionalism analysis, as it implies the gestation of a decision-making level above the state. With regards to global health, however, we would argue that we need to look beyond international law in the strict sense. As we will discuss further below, only WHO received a formal mandate to elaborate, promulgate or implement international law. The World Bank, and the Global Fund cannot formally impose the policies they develop on states; at least in theory states can opt out, or stick to the policies they prefer. In reality, however, the opportunity cost of not adhering to World Bank or Global Fund policy can be very high, in particular for low-income countries that are highly dependent on the international assistance that comes with policy adherence. WHO too often uses policy advice that does not formally bind its member states, but that nonetheless comes with consequences attached to non-adherence. Therefore, our analysis looks beyond international law in the strict sense, to include policy that is binding in the broader sense: policy that states cannot reject without risking serious consequences. In legal jargon, our approach is in line with Klabbers’ definition of “global administrative law”, [30] and Allott’s definition of “new international law” [31].

While the importance of democratic legitimacy is easy to understand, whenever states are actively or passively transferring a substantial part of their decision-making powers to international or global organisations, it is less easy to capture the requirements of democratic legitimacy at the global level. The principle of sovereign equality, on which the United Nations (UN) system is built, leads directly to a ‘one country one vote’ principle, as the yardstick of democratic legitimacy. When assessed from a more cosmopolitan perspective, following a ‘one person one vote’ principle is equally problematic as it accords each inhabitant of a very sparsely populated country more clout on the global stage than each inhabitant of the most populated country. Soon after the foundation of the UN, a committee of 11 scholars, most of them affiliated with the University of Chicago, who felt that the UN was not democratic enough, developed a Preliminary draft of a World Constitution, which proposed the creation of a Federal Republic of the World, with a Federal Convention consisting of “delegates elected directly by the people of all states and nations, one delegate for each million of population or fraction thereof above one-half million” [32]. The idea of a Federal Republic of the World has never been seriously considered by the international community, but the ‘1,000.000 persons one vote’ approach is still useful as an alternative way to measure democratic legitimacy. Meanwhile, the intellectual search for democratic legitimacy at the global level continues, [33–37] and is currently focused on the inclusion of ‘civil society’. As Besson expresses it: “Indeed, once the multilateral and multilevel international political community is understood as a pluralistic community of communities and as a hybrid community of states and individuals, the equivalence of sources and the plurality of specific regimes within international law becomes a democratic requirement” [27]. In other words, to claim democratic legitimacy, global institutions should include civil society representatives in their decision-making bodies. Last but not least, to claim democratic legitimacy a global institution should, in our opinion, not only take decisions in line with the opinion of the people or countries it represents, it should also have the power to ensure that those decisions are implemented by all concerned. Both the ‘demos’ (‘people’ in ancient Greek) and ‘kratos’ (‘power’ in ancient Greek) are essential: a government that is taking a decision to impose a tax on the properties of the wealthiest members of the population, in accordance with the majority opinion, but unable to make the wealthiest people pay the tax, is not a truly democratic government: in the end, the minority decides to pay the tax, or not. As we will discuss further below, this is the main obstacle blocking institutions at the core of the global health governance architecture from claiming democratic legitimacy.

Finally, with regards to human rights law and the realisation of the right to health in particular, we should clarify our understanding of the right to health. Like other international legal scholars, our definition of the right to health flows from the legal basis of the right as enshrined in the 1966 Covenant on Economic, Social and Cultural Rights (Covenant),[38] and expanded upon in the 2000 General Comment 14 issued by the UN Committee on Economic, Social and Cultural Rights (Committee) [39]. The right to health is not a right to be healthy but a legitimate claim to certain freedoms and entitlements, [40] such as the entitlements to water, food, housing and healthcare. It enshrines fundamental human rights principles of participation, accountability, non-discrimination, transparency and shared responsibility: national and international obligations, the latter related to development assistance and cooperation for health. The importance of the concept of ‘core obligations’ – what states need to do to realise minimum essential levels of the right to health-and the existence of international obligations of assistance from wealthier states to states that are unable to live up to their core obligations, are both of vital importance for advancing the right to health for all [41, 42]. When using constitutionalist principles to assess global health governance, we would argue that the realisation of these minimum essential levels, through shared national and international responsibility, is the minimum standard by which achievements, or lack thereof, of global health governance should be assessed, before one can claim that global health governance is or is not constitutional. For reasons explained further below, it is important to highlight that infectious disease control is an essential element of the right to health. It is mentioned explicitly in the Covenant-article 12.2.(c): “The prevention, treatment and control of epidemic, endemic, occupational and other diseases” – and, in General Comment 14 – paragraph 44.(c): “To take measures to prevent, treat and control epidemic and endemic diseases”. However, the core content of the right to health is more comprehensive than infectious disease control. Thus, wheninternational cooperation, or policy, developed by the global institutions at the core of the global health governance architecture, is primarily concerned with infectious disease control this cooperation or policy contributes only partially, and selectively, to the realisation of the right to health.

### III. Applying a global constitutionalism analysis to the core structure of the global health governance architecture

#### III.1. Rule-making authority

##### WHO

Of the three global institutions analysed here, only WHO has a formal mandate to elaborate or promulgate international law. Article 19 of the WHO constitution grants the World Health Assembly (WHA) “authority to adopt conventions or agreements with respect to any matter within the competence of the [WHO]”, “which shall come into force for each Member when accepted by it in accordance with its constitutional processes” [10]. In other words: article 19 of WHO’s constitution is the legal basis for it to prepare and elaborate international law on all health issues, but its member states are free to ratify the resulting conventions or treaties, or not. Article 21 accords the WHA the authority “to adopt regulations” concerning specific international health issues: “(a) sanitary and quarantine requirements and other procedures designed to prevent the international spread of disease; (b) nomenclatures with respect to diseases, causes of death and public health practices; (c) standards with respect to diagnostic procedures for international use; (d) standards with respect to the safety, purity and potency of biological, pharmaceutical and similar products moving in international commerce; (e) advertising and labelling of biological, pharmaceutical and similar products moving in international commerce.” [10] According to article 22, “[r]egulations adopted pursuant to Article 21 shall come into force for all Members after due notice has been given of their adoption by the Health Assembly except for such Members as may notify the Director-General of rejection or reservations within the period stated in the notice” [10]. In other words: articles 21 and 22 of WHO’s constitution are the legal basis for its authority to prepare and elaborate international law on specific health issues, which is binding for all member states, except those who explicitly reject it (which comes at a diplomatic cost).

WHO’s use of this double mandate has been selective. WHO used the mandate enshrined in articles 21 and 22 of its constitution to promulgate the International Sanitary Regulations of 1951, revised and renamed the International Health Regulations (IHR) in 1969, and again-now more thoroughly-revised in 2005 [8]. Five member states made reservations, no member state rejected the revised IHR. WHO has used the mandate enshrined in article 19 of the WHO constitution only once, when it negotiated the Framework Convention on Tobacco Control (FCTC) [43]. Out of 194 WHO member states, 179 are parties to the FCTC.

As mentioned above, WHO’s most ambitious plan for promoting equitable access to essential healthcare, the practical expression of its mandate to ensure the highest possible level of health for all, was the Global Strategy for Health for All by the Year 2000, [12] based on the 1978 Declaration of Alma-Ata [13]. WHO did not ‘translate’ the Global Strategy for Health for All by the Year 2000 into binding international law. Instead, it used non-binding instruments: a WHA resolution in May 1981, [12] followed by a UN General Assembly resolution in October 1981 [44]. With a non-binding instrument, we mean a document like a declaration, resolution or code, in which states agree on a common objective, and on means to achieve that objective, without making a formal commitment to do what it takes to achieve the commitment. As discussed above, we do not consider it appropriate to exclude non-binding instruments from a global constitutionalism assessment of global health governance, as some of them come with important financial rewards, making it very difficult for some countries to reject them. However, WHO’s non-binding instruments do not fit in that category, as WHO was never set up to become a channel of international health financing, and does not have the financial clout to ‘buy compliance’.

WHO made several other attempts to contribute to the realisation of the right to health via non-binding treaties. For example, in 2004, WHA resolution 57.19 requested the WHO Director-General to develop “a code of practice on the international recruitment of health personnel, especially from developing countries” [45]. A footnote in this resolution mentioned explicitly: “It is understood that, within the United Nations system, the expression “code of practice” refers to instruments that are not legally binding.” Nonetheless, some scholars, including Taylor-one of the main proponents of the (binding) FCTC-argue that the WHO Global Code of Practice on the International Recruitment of Health Personnel (Global Code) “incorporates procedural mechanisms to advance implementation that are more potent than those incorporated in the FCTC” and that “[w]hile both the FCTC and the WHO Global Code set forth a shallow substantive framework, the WHO Global Code sets forth a deep legal and institutional framework” [46]. Another example: in 2008, the WHA adopted resolution 61.21 on a “Global Strategy and Plan of Action on Public Health, Innovation and Intellectual Property”,[47] which requested the WHO Director-General “to establish urgently a results-oriented and time-limited expert working group to examine current financing and coordination of research and development”. The Consultative Expert Working Group on Research and Development (R&D): Financing and Coordination (CEWG) that was created recommended that governments begin negotiations on a global medical R&D convention [48]. But even the Chair and the Vice-Chair of the CEWG expressed doubts about the kind of instrument that would fulfil the objectives best: “Some argue that the only way to get agreement on strong and specific enough measures is through soft laws, since hard laws often end with watered down commitments, and that soft law thereby can achieve more”. Although they continue to argue for a “legally binding instrument with clear commitments; a new international convention”, [49] it seems rather unlikely that a convention will be agreed upon in the near future.

##### The World Bank

The World Bank has no formal mandate to elaborate or prepare international law in the strict sense, but it has money to back up its recommendations. Neither SAPs, nor the 1986 ‘Financing health services in developing countries: an agenda for reform’ paper, [50] nor the 1993 ‘Investing in Health’ report, [21] can be considered as international law in the strict sense. However, for poorer countries, the cost of non-compliance was high: not adopting a World Bank and International Monetary Fund (IMF) approved SAP meant being excluded from support from the ‘soft loan arm’ of the World Bank, namely the International Development Association (IDA). In addition, often it also meant dwindling international financial support from many high-income countries that considered the absence of a SAP as a sign of irresponsible governance. As Lee and Goodman emphasise, “a key feature of SAPs was the policy condition to reduce public expenditure on the social sectors including health” [51].

It should be mentioned that, by the end of the 1990s, the World Bank softened its policies. At least in theory, the Poverty Reduction Strategy Papers (PRSPs) that replaced the SAPs are ‘country owned’, developed by the countries themselves. They should therefore be less invasive with regards to the national policy space. Opinions on whether PRSPs are truly different from SAPs vary [52]. What matters, for our analysis, is that as with SAPs, non-compliance with PRSPs comes with a serious cost.

When assessing the rule-making authority of the World Bank, it is also important to recognise that countries that contribute to the World Bank relinquish their discretionary power over the use of the money they provide. In fact, they even give up some discretionary power over the amount of money they provide to the World Bank. The key instrument of World Bank aid is the IDA, and about 70 % of the IDA’s financial resources come from high-income countries via ‘replenishments’ – the high-income economy IDA members are supposed to contribute, more or less in accordance with their Gross Domestic Product (GDP). Weiss describes the process-with regards to the USA-as follows: “IDA replenishments comprise two distinct phases: negotiating the replenishment round and annual contributions. First, the donor nations negotiate the overall amount of a 3 year replenishment, individual donor contributions, and general policy considerations for the round. Following this, each member country seeks annual contributions, typically through its legislative process, to meet their IDA commitments” [53]. And he continues: “the United States is obligated to contribute the amount agreed to at the replenishment” [53]. Thus the World Bank captured some rule-making authority from high-income countries.

##### The Global Fund

The Global Fund, like the World Bank, has no formal mandate to elaborate or prepare international law in the strict sense. Furthermore, it was created with the explicit intention of financing interventions and programs designed at the national level, thus increasing national policy space by increasing available resources [54]. Over the years, however, through its decisions about funding or not funding certain proposals, and several documents guiding the preparation of proposals, the Global Fund has developed its own policy. The cost for poorer countries of not ‘complying’ with Global Fund policy is similar, albeit less severe, than the cost of not complying with World Bank policy: forfeiting substantial amounts of international financing. (With less severe, we mean that the costs of non-compliance with Global Fund policy is limited to forfeiting Global Fund financing, while the cost of non-compliance with the World Bank’s health sector policy could extend beyond the health sector.)

Furthermore, the creation of a global fund with a mandate limited to three infectious diseases is in and of itself a strong engine for health sector development that emphasises infectious disease control. It should be noted that at least some countries wanted the Global Fund to be a global fund for health from the onset [55]. Thus, the implicit decision by the Global Fund Board not to go in that direction can be seen as a global level decision that limits policy space at the national level.

Are the countries, and private entities, that contribute financial resources to the Global Fund also giving up some of their decision-making power? The answer is yes, at least in relation to the money they contribute, they accept that the Global Fund’s decision-making mechanisms determines allocations. But with regards to the size of their contributions, they retain their discretionary power. Like the World Bank-or more precisely, the IDA-the Global Fund is financed through replenishments. The difference between both is subtle, but significant: while contributions to the IDA follow a GDP-based burden-sharing key that has been respected by and large over several replenishments, the Global Fund proposes different scenarios of “illustrative contributions” – pro rata to earlier contributions, based on shares of funding to IDA, based on adjusted Gross National Income (GNI) – which are, at best, informative [56]. Thus, unlike the World Bank, the level of contributions to the Global Fund remains a matter of discretionary choice by the contributing states.

Summary:

All of the three institutions we identified as comprising the core structure of the global health governance architecture (with the Global Fund as an example of GHIs) have been given, or have managed to capture, rule-making authority at a level above the state-level. WHO received a formal mandate to promulgate international law. The World Bank and the Global Fund do not have such a mandate, but they have financial resources that make it difficult for poorer countries to not comply. Further-more, the World Bank has also captured some rule-making authority from wealthier countries, in the sense that it can rely on financial contributions that are based on wealthier countries’ GDP and are thus not entirely discretionary.

#### III.2. Democratic legitimacy

##### WHO

The highest governing body of the WHO is the WHA, where each member state has one vote. For that reason, it is often considered as the most democratic global health institution; according to Harmer, it is “perhaps the *only* global health institution that still retains the vestige of democracy” [55]. However, when assessed from a more cosmopolitan perspective, using a ‘one person one vote’ principle, it is problematic that the inhabitants of small countries have, together, as many votes as the inhabitants of countries with a much bigger population. For example, the 11 million inhabitants of Belgium-the country where both authors live-have one vote (together), and the 316 million inhabitants of the USA also have one vote (together). The voting power of the average inhabitant of Belgium is thus 30 times stronger than the voting power of the average inhabitant of the USA.

Furthermore, as mentioned above, most scholars involved in the search for global democracy now agree that, one way or the other, civil society organisations should be included in the decision-making processes of global institutions. For WHO, the challenge of including civil society in its decision-making processes seems to be a never-ending saga. In 2010, Kickbusch and colleagues suggested the creation of a ‘Committee C’ within WHO, to give both civil society and the private sector a space and a voice [57]. The proposal was not accepted as such, but probably was the trigger for discussions that lead to the adoption of a new “WHO framework on engagement with non-state actors” at the May 2016 WHA meeting. In the words of Director-General Margaret Chan, “[t]he framework on engagement with non-state actors was arguably the most difficult item to negotiate” [58]. It is too soon to assess the impact of this new framework.

However, the main reason why we would be reluctant to confer democratic legitimacy to WHO lies in the second part of the word demo-cracy. A democratic institution should have the power to make all its members or its entire constituency abide by the decisions taken by the majority. The events following the Global Strategy for Health for All by the Year 2000 illustrate WHO’s powerlessness when it comes to implementing a strategy that is widely supported by most poorer and many wealthier states, but frowned upon by a handful of wealthier states. Not only does WHO lack the financial resources to back up its policies, it is itself dependent-and increasingly so-on the discretionary financial support of a handful of wealthier countries [59].

##### The World Bank

The highest governing bodies of the World Bank’s institutions-the World Bank is a common name for the International Bank for Reconstruction and Development (IBRD), International Finance Corporation (IFC), the IDA, and the Multilateral Investment Guarantee Agency (MIGA) – are the Board of Governors and the Board of Directors. Voting rights depend on the number of shares countries acquired: using the same countries as above to illustrate this, the ‘one dollar, one vote’ approach leads to the USA having 10 % of the total voting rights, while Belgium has 1 % of the voting rights. So, at first sight, the World Bank appears as less democratic than WHO from a sovereign equality perspective, but more democratic from a cosmopolitan perspective: the voting rights of the average inhabitant of Belgium are only 3 times greater than the voting rights of the average inhabitant of the USA (as opposed to 30 times greater in WHO). However, our illustration would be incomplete without including poorer countries (with fewer shares). Burundi, a country with the same population as Belgium, has 0.2 % of the voting rights. Nigeria, the country with the highest population in Africa – 17 times as many inhabitants as Belgium – has 0.4 % of the voting rights. The voting rights of the average inhabitant of Belgium are 40 times greater than s the voting rights of the average inhabitant of Nigeria. Even from a cosmopolitan perspective, the democratic legitimacy of the World Bank is highly questionable.

Civil society has no voting rights in the World Bank. Since 2008, together with the IMF, the World Bank organises a Civil Society Policy Forum at the same time as its biannual Board of Governors meetings [60]. Whether this forum really influences World Bank policy remains to be seen, but we nonetheless consider it an interesting development. For example, in October 2016, Oxfam, the ONE Campaign, and Save the Children, used the Civil Society Policy Forum to challenge the World Bank on its ambiguity when it comes to financing UHC [61].

When it comes to the question of possessing the power to make its members abide by decisions taken together, the World Bank is obviously the most ‘cratic’ global health governance institution-we are reluctant to call it demo-cratic because of the distribution of voting rights. The World Bank acquired financial autonomy to a large extent, partly because of its own resources (reimbursement of loans), and partly because of the standing practice of GDP-based contributions to the IDA replenishments. The World Bank has far less to fear than WHO (and the Global Fund, as will be discussed further below), that wealthier countries could turn their backs, and close their wallets, if it pursues f policies these countries dislike. But then again, the chance that the World Bank adopts and promotes policies disliked by wealthier countries is small, given the distribution of voting rights.

##### The Global Fund

The Board of the Global Fund is the highest governing body of the Global Fund. On the Global Fund Board, countries are grouped together into constituencies: half of them represent the providers of international financing, the other half represent the recipients or potential recipients of international financing. (Several countries that have either indicated that they will no longer apply for Global Fund funding, or that are no longer eligible because of the Global Fund’s policies, remain on the ‘recipient side’: for example, Brazil and China.) Thus about 20 high-income countries have as many votes as about 170 low-and middle-income countries: not quite democratic from an sovereign equality perspective. From a cosmopolitan perspective, we will use the same countries as above to illustrate the situation. The USA has its own seat on the Global Fund Board, with one vote, or 5 % of all the votes. Burundi is a member of the Eastern and Southern Africa constituency, together with 19 other countries, so we could say that it has 5 % of a vote, or 0.25 % of all the votes. Nigeria is a member of the West and Central Africa constituency, together with 21 other countries, so we could say that it has 4.5 % of a vote, or 0.23 % of all the votes. Belgium is a member of the constituency with Italy, Portugal and Spain, which includes the European Commission (EC) as well, which makes it difficult to calculate the voting rights-should we consider the EC as one entity, or as the representative of all members of the European Union (of which most would then be represented twice)? If we consider Belgium as one of five members of this constituency, it has 20 % of its constituency’s vote, or 1.25 % of all votes. When we then divide the voting rights of each of these four countries by the number of inhabitants, we can say that the average inhabitant of Belgium has 0.000 000 039 % of the votes, the average inhabitant of Burundi has 0.000 000 012 % of the votes, the average inhabitant of Nigeria has 0.000 000 011 % of the votes, and the average inhabitant of the USA has 0.000 000 016 % of the votes. The biggest difference here is between Belgium and Nigeria: the voting rights of average inhabitant of Belgium are about 4 times greater than the voting rights of the average inhabitant of Nigeria: far less than the biggest difference at WHO (where the voting rights of the average inhabitant of Belgium are about 30 times greater than the has voting rights of the average inhabitant of the USA) or at the World Bank (where the voting rights of the average inhabitant of Belgium are about 40 times greater than the voting rights of the average inhabitant of Nigeria). This is, of course, a purely mathematical approach, with little political meaning, but we nonetheless find it interesting to see how a constituency approach can find a middle ground, in between the sovereign equality and cosmopolitan approaches.

More important, in our opinion, are the non-state constituencies of the Global Fund: foundations have a vote on the board, as does the private sector, civil society of developed countries, civil society of developing countries, and communities affected by the three diseases. Whether the inclusion-with voting rights-of these non-state actors increases or decreases the democratic legitimacy of the Global Fund is food for a separate debate. Some would argue that the private sector already has too much indirect influence, without being formally included in matters of global public governance. Even the inclusion of civil society raises questions. As Tallberg and Uhlin note (on civil society and international organizations in general, not about the Global Fund in particular): “[a]mong civil society actors, well-organized and well-funded [Non-Governmental Organizations] tend to be overrepresented, whereas marginalized groups from developing countries tend to be highly underrepresented” [37]. We presume that people with an inclination to the sovereign equality principle look rather unfavourably upon the Global Fund’s democratic legitimacy, while people with an inclination towards the cosmopolitan approach will notice the attempt to create “a plurality of specific regimes”, in line with Besson, [27] with a benevolent eye.

However, to complete the assessment of the Global Fund’s democratic legitimacy, we cannot ignore that it is and remains, even more then WHO, dependent on discretionary financial support from the wealthier countries. In theory, the Board of the Global Fund could decide to expand its mandate and to become a global fund for health, as some countries wanted it to be from the beginning [55]. If all constituencies would agree, except, for example, Australia and the UK (one vote together), the USA, France and Germany (one vote each), all the others would still have a majority, on the ‘recipient side’ and on the ‘contributor side’. In reality, it would be sufficient for two or three of these countries to warn that they would end their contributions, to make all the other countries abandon their attempt.

Summary:

In our opinion, none of the three institutions we identified as comprising the core structure of the global health governance architecture can claim democratic legitimacy. WHO appears as the most democratic from an equal sovereignty perspective, the Global Fund appears as the most democratic from a cosmopolitan perspective, but both lack the financial autonomy that would allow them to pursue policies that are not approved by a handful of wealthier countries. The World Bank has that autonomy, at least to some extent, but its ‘one dollar one vote’ distribution of voting rights mechanism rules it out from possessing democratic legitimacy.

#### III.3. Contribution to the realisation of the right to health

##### WHO

As mentioned above, the WHO was created in 1946 with a multi-pronged mandate, including a mandate to promote “the attainment by all peoples of the highest possible level of health” and a mandate to promote the “control of disease, especially communicable disease” [10]. Until recently, this duality was reflected in the way the WHO presented itself on its website: “In the 21st century, health is a shared responsibility, involving equitable access to essential care and collective defence against transnational threats.” During the first decades of its existence, until the mid-1970s, the WHO’s focus was on disease control [2–5]. This focus can be explained by the historical legacy of the WHO, by its technical capacities at the time, but maybe also by the reality that disease control reflects the shared interest of all its members best: advancing equitable access to essential care is important mostly to poorer countries, whereas infectious disease control is important for all countries. Furthermore, infectious disease control is an essential element of the realisation of the right to health, but the realisation of the right to health demands much broader efforts.

WHO’s focus changed when Halfdan Mahler became Director General. Davies calls this the ‘humanitarian’ phase of WHO, [5] we would rather call it a turn to ‘social justice’. Mahler himself remembers it as such, when commenting on the Declaration: “The 1970s was a warm decade for social justice. That’s why after Alma-Ata in 1978, everything seemed possible” [62]. The Declaration, [13] and the WHO Global Strategy for Health for All by the Year 2000,[12] can be viewed as marking the zenith of the social justice phase of WHO, and perhaps they constitute the most important contribution of WHO to the realisation of the right to health, as we understand it. Youde calls them “truly revolutionary”, while emphasising the shift from national to international or global responsibility they entailed: “Up to this point, health care has generally been the sovereign domain of states”, and “[b]y promoting the Alma-Ata Declaration and Health for All by 2000, the assembled delegates sought to have states declare that public health was no longer simply a concern for national governments” [3]. The zenith of the social justice phase of WHO, however, also marked the beginning of the demise of WHO’s position as the leader of global health governance. As noted above, some wealthier countries, the USA first and perhaps foremost, were not pleased with what they perceived as WHO advancing a political agenda, and started enabling the World Bank to become the leader in global health governance [17]. The next phase of the WHO, which Davies calls ‘neoliberal’, [5] can perhaps best be understood as an attempt to find a relevant role in the shadow of the World Bank: “WHO had to quickly adapt its health packages to help secure the deliverables that the World Bank was expecting from health care programmes” [5].

Since the end of the 1990s, WHO has been trying to reassert its role as the leader in global health governance in what has become a rapidly changing landscape, in large part due to the creation of many new GHIs, including the Global Fund [5]. The promotion of Universal Health Coverage (UHC) as the legitimate heir to PHC can be understood as a return of the WHO towards its earlier social justice phase. However, whereas the 1982 Plan of Action for Implementing the Global Strategy for Health for All emphasised states’ responsibility to finance PHC-states will “mobilize all possible financial and material resources” [63] – the present WHO strategy on UHC emphasises risk pooling or avoiding out-of-pocket expenditure: it does not seem to matter whether the financing is public or private, as long as it is not out-of-pocket. From a right to health perspective, the responsibility to finance UHC is a public responsibility. Furthermore, when it comes to shared-national and international-responsibility, the WHO guidance on UHC is far less explicit than its guidance on primary health care used to be. The 1982 Plan of Action for Implementing the Global Strategy for Health for All explicitly included a provision on shared responsibility providing that all countries would “mobilize all possible financial and material resources”; that “Member States requiring external funds in addition to their own resources will clearly identify those needs”; and that “[d]eveloped countries will transfer resources to developing countries that are ready to devote substantial additional resources to health, and will review the nature of these transfers with a view to complying with the needs of the Strategy” [63]. WHO guidance on UHC only mentions that some low-income countries will need assistance [42].

WHO could try to ‘translate’ its policy on UHC into international law, on the basis of article 19 of its constitution, [10] and include specific targets for domestic and international financing. UHC, including targets for domestic and international financing, is at the core of Gostin’s proposal for a Framework Convention on Global Health [64]. Keeping in mind what happened after the Global Strategy for Health for All by the Year 2000, we can understand WHO’s reluctance.

##### The World Bank

As mentioned above, the World Bank’s impact on global health-and its contribution to the realization of the right to health-cannot be dissociated from its involvement with SAPs. Several scholars argue SAPs obliged developing countries to abandon the comprehensive PHC care approach and to accept the selective PHC approach,[17, 18] although some argue that “empirical studies present both positive and negative outcomes of adjustment programs [on health]” [65]. In any case, the 1987 World Bank report on ‘Financing Health Services in Developing Countries’ explicitly mentioned that “in most countries the general budget stringency makes it difficult to argue for more public spending”, and did not mention international financing as a solution to relax this stringency [50]. This report also explicitly promoted the introduction of user fees, making an exception only for health services “with largely public benefits”, namely infectious disease control, because “[c]onsumers are almost always willing to pay directly for health services with largely private benefits”, but “are generally reluctant to pay directly for programs and services which benefit society or communities as a whole” [50]. The logic behind that recommendation was reaffirmed in the World Bank’s 1993 World Development Report on ‘Investing in Health’ [21]. As noted above, in this report, the World Bank introduced a separation between “public health”, “essential clinical services”, and “the rest of the health system”, and recommended increasing public investment in public health measures focused on infectious disease control, recommended that “the rest of the health system” be financed privately, and remained ambiguous on the financing of “essential clinical services” [21]. Coincidently or not, the ensuing health sector reforms resulted in international assistance being used predominantly for what governments decided to finance publicly at the national level, namely infectious disease control. In our opinion, the World Bank’s health sector reform policies of the 1980s and 1990s, in combination with SAPs, constituted violations of the right to health [66].

By the end of the 1990s, it became obvious that the World Bank’s health sector reform strategy, emphasising national self-reliance, had gone too far. While “[u]ntil 1990 the global effort to immunise the world’s children had been a remarkable success story”, [23] the health systems of low-income countries proved too weak to integrate immunisation programs previously financed by the international community. These health systems were also ill prepared for the pandemic of HIV/AIDS. Against this backdrop, the new GHIs were created, including the Global Fund.

In recent years, the World Bank has become a supporter of UHC. This can be understood as a departure from its earlier health financing strategies for poorer countries, or as an explanation for WHO’s ambiguity on public or private responsibility for financing UHC. One can easily imagine a form of UHC that is entirely in line with present (ambiguous) definitions of UHC and the World Bank’s 1993 World Development Report on ‘Investing in Health’: [21] one in which infectious diseases control is financed through public resources, while the financing of all other elements of UHC is left to private health insurance.

##### The Global Fund

According to Davis, the “Global Fund’s mandate-to direct resources to support the fight against HIV, TB, and malaria-is grounded in a human rights commitment: It supports governments in their obligation under the International Covenant on Economic, Social and Cultural Rights (ICESCR) and the Universal Declaration of Human Rights (UDHR) to progressively realize the right to the highest attainable standard of health” [67]. We agree that the Global Fund contributes to the realisation of an important part of the right to health, in a way that neither WHO nor the World Bank does: WHO because it does not have the resources to do so (as it was not designed to be a channel for international financing); the World Bank because it remains reluctant to allow poorer countries to use international financing for recurrent expenditure, as it considers international financing as unreliable in the long run [68]. This attitude fails to appreciate that international financing of healthcare in poorer countries is an integral part of the right to health, and, it is also somewhat ironic, given that the World Bank’s GDP-based replenishments put it in an excellent position to provide reliable international financing. In any case, if the Global Fund had adopted the same approach, it would not have financed AIDS treatment-recurrent par excellence. Therefore, the Global Fund is an embodiment of ‘shared responsibility’, which is a key element of the right to health.

However, from a right to health perspective, it is not easy to justify the Global Fund’s mandate being limited to three diseases. Ever since the creation of the Global Fund, attempts have been made from within the Secretariat and members of the Board to broaden the mandate, [55, 69] or at least to increase the Global Fund’s support to wider health systems,[70] but the result of these attempts has been disappointing.

Summary:

The Global Strategy for Health for all by the Year 2000 would have allowed WHO to claim a leadership award in realising the right to health, if WHO would have had the financial clout and autonomy to follow through on implementation. The Global Strategy for Health for all by the Year 2000 may have had a positive impact in middle-income countries, freeing them from reliance on World Bank and IMF support, but was derailed by SAPs and health sector reform policies of the World Bank. The World Bank’s track record in realising the right to health is weak. The Global Fund can be seen as an embodiment of ‘shared responsibility’, which is a key element of the right to health. But its mandate, limited to fighting three infectious diseases, stands in the way of it claiming a leadership award in realising the right to health.

## Discussion

Health indeed appears as “one of the most dynamic realms of global governance today”, [3] as Youde argues. But the red thread that runs through our global constitutionalism analysis is that the “different ways in which the international community conceptualizes its responsibility for addressing cross-border health concerns”, [3] invariably lead us towards diseases that cross borders, not to global health inequalities that should be a “cross-border health concern” in the moral sense.

Transnational rule-making authority has been conferred to global health governance. WHO, the World Bank, and the Global Fund, have received or captured part of the policy space that used to be confined to national governments. While WHO has a mandate to elaborate and promulgate international law, it has used it mainly for infectious disease control: the IHR. The only exception so far is the FCTC, which also addresses a cross-border health concern that affects high-income countries directly. The World Bank and the Global Fund acquired their rule-making authority from the financial incentives they use to back up policy recommendations.

In our opinion, none of the three institutions at the core structure of the global health governance architecture can claim democratic legitimacy. WHO is governed in accordance with the ‘one country one vote’ principle, but finds itself at the mercy of a handful of wealthier states. These states accepted WHO as the leader of global health governance as long as its work was focused on infectious diseases. When the WHO strayed from this focus, these states empowered the World Bank, in particular through increased funding, to assume the global lead, and the World Bank promoted health sector reform skewed towards infectious diseases control. When that proved ineffective, GHIs were created and funded to support infectious disease control. Applying constitutional principles to global health governance leads us to look for efforts to realise the right to health more broadly, but we were disappointed: perhaps an illustration of what Macdonald and Johnston had in mind when warning that global constitutionalism “promotes dangerously seductive “over-expectations” [8].

Thus, the only form of constitutionalism global health governance can claim today would have the characteristics of a ‘mixed constitution’, as defended in 1642 by King Charles I of England, Scotland and Ireland, in response to the XIX Propositions of his defiant parliament. “There being three kindes of government amongst men, Absolute Monarchy, Aristocracy, and Democracy, and all of these having their particular conveniences and inconveniences”, [71] or so argued Charles I, a “mixture” of these “kindes of government” would give England “the conveniences of all three, without the inconveniences of any one”. Charles I then used his monarchic powers, the House of the Lords (then called ‘House of Peers’) and the House of Commons as he saw fit-probably in the way most convenient for his purposes. It does not require a wild imagination to see a similar power game in global health governance today: the USA, and a handful of wealthier countries, use WHO when they need it to create buy-in from all other countries for infectious disease control; they use the World Bank when they do not need consent from other countries because they can buy compliance, or they create new institutions.

## Conclusion

According to Peters, global constitutionalism helps to “uncovers legitimacy deficits” in the process of global constitutionalisation, and also “suggests remedies” [7]. Our global constitutionalism analysis has indeed uncovered legitimacy deficits. Did it also suggest remedies? We think it did. It revealed that the lack of financial clout to back up its policy stands in the way of WHO’s claim to democratic legitimacy, while the Global Fund faces a similar problem because of its dependence on discretionary contributions from a handful of wealthier countries. Any real remedy would have to address the underlying financial problem. That is why we, in earlier work, insisted that the Global Fund could only transform itself into a global fund for health if contributions were made mandatory-in line with international obligations arising from the right to health [72]. Gostin’s proposal of a FCGH is even more ambitious, as it would not only clarify national and international responsibilities for health financing in poorer countries, it would also create financial autonomy for WHO [64]. If the FCGH would also confer jurisdiction to the International Court of Justice (ICJ) – meaning that in the event of a conflict arising from the implementation of the FCGH, states would be able to seek a ruling from the ICJ-we would have the classical tripartite division (or separation) of powers at the global level, with WHO possessing the legislative (law-making) power, the Global Fund (with a wider mandate) possessing the executive power, and the ICJ the judiciary power.

Unfortunately, our global constitutionalism analysis also confirms Nagel’s doubts about the feasibility of global health justice, arguing that “[w]e are unlikely to see the spread of global justice in the long run unless we first create strong supranational institutions that do not aim at justice but that pursue common interests and reflect the inequalities of bargaining power among existing states” [73]. This suggests we may have to put up with a global health governance structure that is controlled by a handful of powerful and wealthier countries for the foreseeable future. Furthermore, the current political climate-with global health security at the top of the global health governance agenda, financial austerity, and a turn towards nationalism in several parts of the world-seems to be far from an ideal moment for an attempt to make global health governance more democratic (whether under the sovereign equality or cosmopolitan approach).

But we could also read Nagel in a way that recognises that we have had strong supranational institutions that pursue common interest (and reflect inequalities of bargaining power) for long enough, and that they have grown strong enough to be challenged on their democratic illegitimacy and failure to realise the right to health comprehensively. Furthermore, if one is of the opinion that substantial modifications to any global governance regime usually come from tension and struggle, rather than from harmony and cooperation, the present time could be an ideal moment for ‘a new global health deal’.

## Abbreviations

CEWG: Consultative expert working group on research and development (R&D): financing and coordination
EC: European commission
FCGH: Framework convention on global health
FCTC: Framework convention on tobacco control
GAVI: Global alliance for vaccines and immunisations
GDP: Gross domestic product
GHI: Global health initiative
Global Code: Global code of practice on the international recruitment of health personnel
Global Fund: Global fund to fight AIDS, tuberculosis and Malaria
GNI: Gross national income
ICJ: International court of justice
IDA: International development association
IHR: International health regulations
PHC: Primary health care
PPP: Public private partnership
SAP: Structural adjustment programme
UHC: Universal health coverage
WHA: World health assembly
WHO: World health organization
